# Gut–Brain Axis in Regulation of Blood Pressure

**DOI:** 10.3389/fphys.2017.00845

**Published:** 2017-10-25

**Authors:** Tao Yang, Jasenka Zubcevic

**Affiliations:** Department of Physiological Sciences, College of Veterinary Medicine, University of Florida, Gainesville, FL, United States

**Keywords:** gut microbiota, immune system, autonomic nervous system, butyrate, blood pressure

## Abstract

Hypertension (HTN) is an escalating health issue worldwide. It is estimated that 1.56 billion people will suffer from high blood pressure (BP) by 2025. Recent studies reported an association between gut dysbiosis and HTN, thus proposing interesting avenues for novel treatments of this condition. The sympathetic nervous system (SNS) and the immune system (IS) play a recognized role in the onset and progression of HTN, while reciprocal communication between gut microbiota and the brain can regulate BP by modulating the interplay between the IS and SNS. This review presents the current state of the science implicating brain-gut connection in HTN, highlighting potential pathways of their interaction in control of BP.

## Hypertension (HTN)

Over the past few decades, HTN has become the most prevalent condition seen in primary care (Mozaffarian et al., 2015), and is the highest modifiable risk factor for cardiovascular disease (CVD) and stroke (Egan et al., 2010). Data from National Health and Nutrition Examination Survey (NHANES) indicate that prevalence of HTN in adults over 20 years was estimated to be 34.0% from 2011 to 2014, in contrast to 67.2% among those over 60 years of age (Benjamin et al., 2017). What is the most alarming is that data generated in NHANES surveys in 2011 to 2012 revealed that the prevalence of high BP was 1.8% among boys and 1.4% among girls aged 8–17 (Benjamin et al., 2017). This underscores the concerns that accompany HTN and its prevalence in younger patients. Higher BP in early adulthood has been associated with high risk of for all-cause mortality, including CVD and coronary heart disease (CHD)-associated mortality. Overall, compared with dietary, lifestyle, and metabolic risk factors, high BP is the leading cause of death in women and the second-leading cause of death in men (Heidenreich et al., 2011).

The pathophysiology of HTN has been intensively investigated, and quite a few factors that contribute to the pathogenesis of HTN are identified. They include, but are not be limited to, uncontrolled activation of immune system (IS) (Singh et al., 2014), overactive sympathetic nervous system (SNS) (Mancia and Grassi, 2014), dampened parasympathetic nervous system (PNS), dysregulation in the renin-angiotensin system (RAS) (Aroor et al., 2013; Kamide, 2014; Cabandugama et al., 2017), endothelial dysfunction (Mendizábal et al., 2013), genetic mutations (Jones et al., 2017; Li et al., 2017b), and diverse environmental factors (Kulkarni et al., 1998; Hamano et al., 2012). In the current review, we focus on the role of IS, SNS, PNS and gut microbiota as an environmental factor in gut-brain axis in the regulation of blood pressure (BP).

## Neurogenic components of treatment-resistant HTN

Treatment-resistant HTN is characterized by uncontrolled high BP that persists despite the combined use of three or more antihypertensive agents of different classes, one of which is a diuretic (Acelajado and Calhoun, 2010). The prevalence of treatment-resistant HTN is estimated to be between 15 and 20% among the hypertensive patient population. Resistant HTN is associated with several factors that include excessive dietary sodium retention secondary to chronic kidney disease (CKD)(Borrelli et al., 2013); obesity (Lohmeier and Iliescu, 2013); prescription drugs (Faselis et al., 2011); heavy alcohol consumption (Pimenta et al., 2008) and obstructive sleep apnea (OSA) (Khan et al., 2013), among others. Overactive SNS is present in all the aforementioned conditions, suggesting a significant role in the pathophysiology of treatment resistance. In addition to chronically elevated SNS activity (Tsioufis et al., 2011) accompanied by norepinephrine (NE) spillover (Tsioufis et al., 2011), dampened parasympathetic activity (Masuda, 2000) is also among several common characteristics of resistant HTN, indicating a neurogenic component that contributes to the initiation, maintenance and progression of HTN. Increasing evidence also suggests that, coupled to autonomic dysfunction, treatment-resistant HTN is accompanied by a chronic low-grade inflammatory profile that facilitates end-organ damage and perpetuates the hypertensive state (Grassi et al., 2011), suggesting a close link between SNS and the IS.

## Role of the autonomic nervous system (ANS)

Environmental cues are perceived by the CNS via the peripheral nervous system afferents. The CNS processes the afferent inputs and organizes the efferent outputs into behavioral and other physiological responses (Bienenstock et al., 2015). In this way, the autonomic nervous system (ANS) involuntarily regulates host physiological homeostasis. The two branches of the ANS, the SNS and parasympathetic nervous system (PNS), cooperate closely to regulate the visceral organs antagonistically, though synergistic regulation also exists (Wehrwein et al., 2016). In the CNS, the central cardioregulatory autonomic centers are located in the hypothalamus and brainstem. Physiologically, ANS efferents modulate the cardiovascular functions and BP in several ways (Wehrwein et al., 2016): (1) sympathetic regulation of heart rate (HR) and vasomotor tone; (2) sympathetic regulation of the endocrine renin angiotensin system (RAS); and (3) parasympathetic regulation of HR. In neurogenic HTN, imbalance in ANS in animal models and human patients (Narkiewicz et al., 2005; Santisteban et al., 2013; Mancia and Grassi, 2014; Zubcevic et al., 2014) leads to over-activation of sympathetic drive, spillover of NE, and peripheral and central inflammation (Mancia and Grassi, 2014; Santisteban et al., 2015). An important aspect is mediated via the stress response pathways (Ulrich-Lai and Herman, 2009), involving the hypothalamus-pituitary-adrenal (HPA) axis and several hormones that uphold the appropriate reactions to perceived threats. Chronic stress continuously activates the HPA axis, resulting in persistent release of glucocorticoid hormone, cortisol (human) or corticosterone (rodent), which exerts its BP-raising ability through its negative effects on vasodilation, and positive effects on RAS (Singh et al., 2011). In addition to the SNS, as mentioned above, the PNS also contributes to the regulation of BP via parasympathetic (vagal) pathway, resulting in the modulation of cardiac output and HR.

## Gut microbiota and the IS

In recent years, gut microbiota has been linked to the initiation and progression of numerous diseases and conditions, including intestinal disorders (Carding et al., 2015) CNS conditions (Mangiola et al., 2016) and various systemic diseases (Chow et al., 2010; Yang et al., 2015). Gut, as the largest immune organ in the body, harbors trillions of bacteria. The numbers of microorganisms within the gastrointestinal (GI) tract in humans are approximately 10 times that of somatic cells in the human body. Moreover, the number of genes the gut microbiota possess exceeds 100 times more than the genes in humans (Kurokawa et al., 2007). Thus, the gut microbiota is a significant variable in how an organism interfaces with and responds to its environment.

The continuous interaction between microbiota and the gut effectively regulates the physiological homeostasis within the gut locally, as well as in the host systemically. Intestinal mesenteric lymph system, also known as Gut-associated lymphoid tissue (GALT), has features of anatomically compartmentalized structure where immune responses are initiated and immune cells are educated. GALT is an interface between the blood and the intestinal lymph fluid, and supplies activated immune cells to intestinal epithelium and lamina propria, where they interact with gut microbiota (Jandhyala et al., 2015). Even in the absence of disease, vast numbers of lymphocytes and other immune effector cells residing across the gut tissue to react to and tolerate gut microbiota. Therefore, the intestinal microbiota plays a critical role in determining the level of immunologic outcomes of various signaling events in host cells. It is inevitable that the intestinal and systemic homeostasis are tightly controlled by regulatory immune mechanisms, which are established by interactions between trillions of microbes, microbial gene products and pattern recognition receptors (PRRs). Disruption of this balance by inimical signals has significant consequences that may result in a vast number of diseases, as previously described (Yang et al., 2013).

To date, a group of commensal bacterial genera have been identified and intensively investigated, including *Lactobacillus, Clostridium, Bifidobacterium, Bacteroides, Streptococcus*, and *Enterobacterium* (Yang et al., 2015; Donaldson et al., 2016). Recent findings from The Human Microbiome Project showed that thousands of microbes inhabit the gut of the human population, with a high degree of variation in composition between individuals (Consortium, 2012). Despite this variation among individuals, the microbial genes involved in the basic up-keep of metabolic activities are functionally similar between individuals (Consortium, 2012).

Gut dysbiosis is generally characterized by a decrease in microbial population diversity and stability, and blooms in certain harmful bacteria (Zeng et al., 2017). The metabolic network within the host harboring dysbiotic microbes can also be altered *in situ*, resulting in insulin resistance and abnormal levels of short chain fatty acids (SCFAs) (Gao et al., 2009; Machiels et al., 2014), among other metabolic disturbances. Inflammatory bowel diseases (IBD), for example, is associated with chronic intestinal inflammation and disruption of the gut barrier has also been partially attributed to gut dysbiosis (Tamboli et al., 2004).

In addition to the role of prebiotics in promoting growth of certain beneficial bacteria (typically *Bifidobacterium* and *Lactobacillus*) (Kootte et al., 2012), and their role in reducing pathological gut leakiness and inflammation (Ulluwishewa et al., 2011), several probiotics have been evaluated in clinical trials in relation to BP regulation. A meta-analysis of 9 randomized trials showed a significant decrease in both the systolic BP (SBP) and diastolic BP (DBP) in patients who consumed a daily dose of ≥10^11^ CFU of *Lactobacillus helveticus* (Khalesi et al., 2014). These studies suggest that gut microbiota play an important role in the control of BP homeostasis and that the correction of gut dysbiosis by probiotics may be beneficial for BP control.

## Communication between the gut and the brain

**Figure 1:**
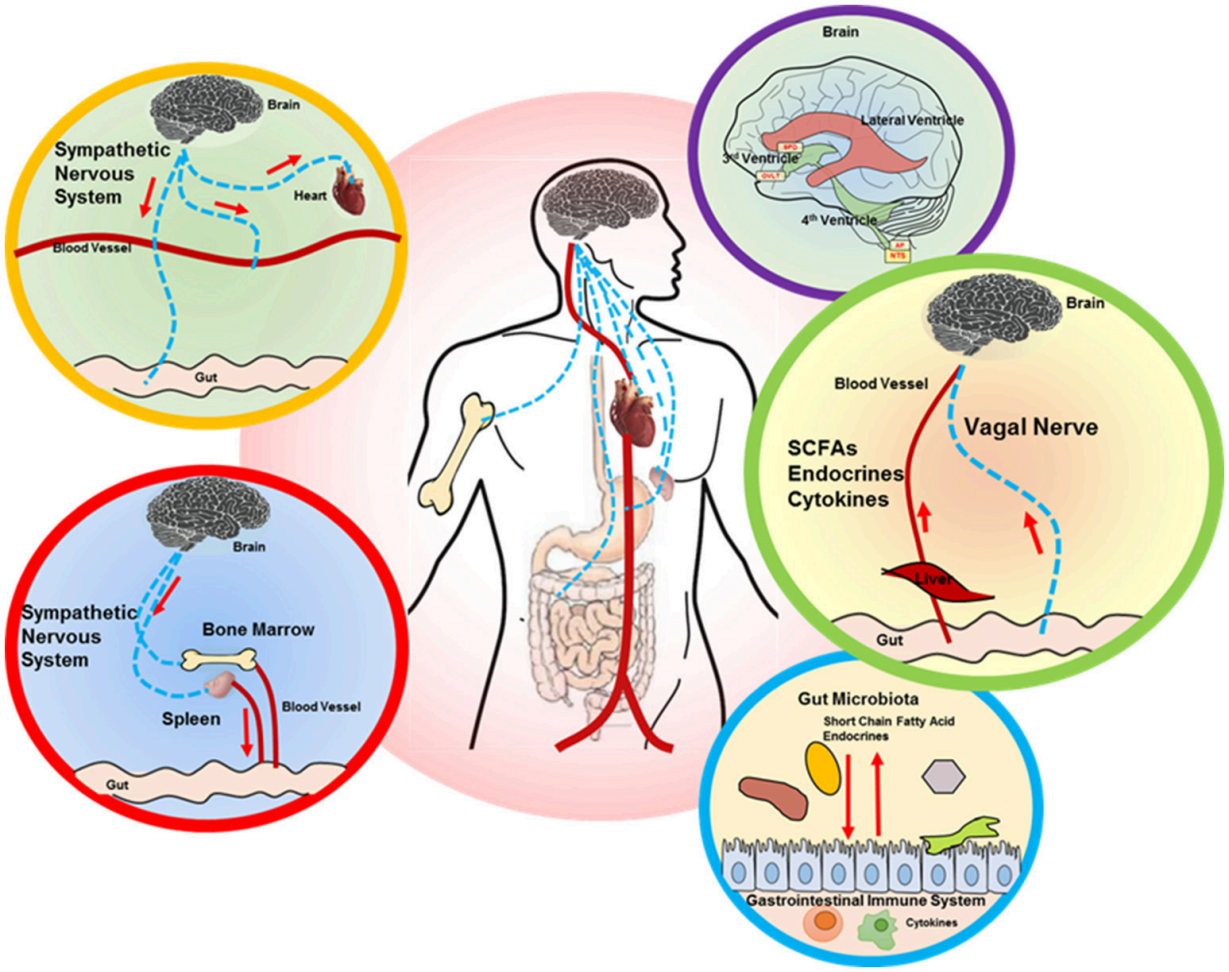
Proposed brain-gut axis in hypertension. A number of signaling mechanisms connect the gut and brain, including the following: (i) descending autonomic innervation of cardiovascular and GI systems (yellow and red circle outlines) and sympathetic regulation of the immune system (red circle outline), which also impacts the gut (red and blue circle outlines); (ii) ascending connections, including circulating factors (SCFAs, endocrines, cytokines) that are perceived by the brain circumventricular organs (CVOs), while vagal signaling from the gut is processed in the NTS (purple and green circle outlines). The interaction between gut microbiota and GI system is shown in the blue circle outline. SCFA, short chain fatty acid; SFO, subfornical organ; OVLT, organ vasculosum of lamina terminalis; AP, area postrema; NTS, nucleus tractus solitarius.

The gut-brain axis involves bidirectional communication between the CNS and the enteric nervous system and gut commensals (Cryan and Dinan, 2012; Bienenstock et al., 2015). The mechanisms behind the emerging gut-brain axis are still not completely clear, but there are several tantalizing hypotheses, which include the role of the IS, bacterial metabolites, vagal afferent pathway and endocrine effects (Figure 1). It is also important to emphasize that these variables likely interact with each other to maintain homeostasis.

### Role of the IS in gut-brain axis

#### IS signaling to the CNS

The CNS responses can be activated via circumventricular organs (CVOs) during systemic inflammation (Johnson and Gross, 1993), as demonstrated in some intestinal disorders (Matteoli and Boeckxstaens, 2013). CVOs are specialized structures lacking the blood brain barrier (BBB), thus allowing direct communication between brain parenchyma and peripheral fluids. As a result, these highly-vascularized CVOs are able to monitor hormonal and cytokine changes in circulation (Akrout et al., 2009; Krause et al., 2011). In addition, vagal afferent pathway also mediates the signaling from IS to CNS, which will be discussed in the vagal pathway section.

#### CNS regulation of the IS

One of the ways through which CNS communicates with IS is via the ANS. Sympathetic innervation exists in both primary (bone marrow, thymus) and secondary (spleen, lymph nodes, mucosa-associated lymphoid tissue) lymphoid organs. NE released from the terminals of sympathetic postganglionic neurons binds to the adrenergic receptors expressed on both innate and adaptive immune cells. The adaptive immune cells respond to SNS cues predominantly via the b2-ARs (Lorton and Bellinger, 2015), and stimulation of b2-ARs on these immune cells modulates diverse aspects of immune cell functions. In the bone marrow, hematopoietic stem cells (HSC) receive direct SNS input via the ARs expressed on the cell surface. This largely physiological response of the IS to the sympathetic tone is beneficial in mobilization of hematopoietic stem and progenitor cells (HSPC) during the “active” period of a day, in anticipation of possible infection and injury (Hanoun et al., 2015). However, this response may become pathological when SNS is chronically activated, regardless of time, such is the case in HTN. In HTN, increased activity of the femoral sympathetic nerve is associated with a significant elevation in NE in the bone marrow, leading to an overactive IS in the SHR (Zubcevic et al., 2014). In the secondary lymphoid organs, sympathetic nerves travel along with the local vasculature and associated connective tissue, and form neuroeffector junctions with the immune cells in the lymphoid parenchyma. GALT is also innervated by sympathetic nerves that extend from the vascular beds in the gut (Bellinger and Lorton, 2014). Interestingly, sympathetic drive avoids the innervation of germinal center where the differentiation and maturation of B cells occurs (Popper et al., 1988), though it is known that B cells can be modulated by the substances released from sympathetic terminals (Pongratz and Straub, 2010). Interestingly, the effects of adrenergic signaling on the immune cells have been implicated in both anti-inflammatory and pro-inflammatory responses (Lorton and Bellinger, 2015), depending on the level of activation of specific immune cells and the stage of the disease (Lorton and Bellinger, 2015).

In addition, the PNS also plays an important role in the regulation of IS. Electrical vagal stimulation experiments demonstrated attenuation of systemic inflammatory responses to endotoxin by reducing the pro-inflammatory TNF responses, but not the anti-inflammatory IL-10 (Borovikova et al., 2000). In subsequent mechanistic investigations, Wang et al. demonstrated that the nicotinic acetylcholine receptor alpha 7 (α7nAChR) present on macrophages is an essential regulator of the anti-inflammatory effects resulting from vagal nerve stimulation (Wang et al., 2003). Therefore, temporary activation of the vagal nerve leads to the release of anti-inflammatory acetylcholine that binds to α7nAChR^+^ macrophage and suppresses the production of pro-inflammatory cytokines (Báez-Pagán et al., 2015). However, chronic inflammation, including that observed in HTN, is associated with attenuation of both afferent and efferent vagal responses (Kentish and Page, 2015), indicating its deleterious effect on vagal reflexes. From this, it is tempting to propose that afferent vagal signaling from the gut may alter the profile of immune cells via modulation of efferent cholinergic tone, thus reducing mucosal inflammation and maintaining the gut homeostasis (Matteoli and Boeckxstaens, 2013).

Therefore, the observed deregulation of both SNS and PNS in HTN may contribute to the exaggerated inflammatory responses in HTN. Any alterations in one or both of these pathways can be involved in the pathophysiology of HTN.

#### Immune responses within the CNS

The brain has the means to generate a local immune response, and this defense mechanism primarily involves glial cells. It has been shown that excessive or sustained activation of central immunity by systemic stimuli results in an imbalance, and even damage, to the neurons that can lead to neuroinflammation and neurodegeneration (Hoogland et al., 2015). Chronic neuroinflammation within the brain cardioregulatory regions reportedly leads to dysfunction of SNS and subsequent elevation in BP (Schlaich et al., 2004). Two major glial cell populations, microglia and astroglia, constitute 5–20% and 20–40% of total glial cell population in the CNS, respectively. Under normal physiological conditions, microglia react to environmental antigens, clear apoptotic cell debris, and maintain the homeostasis of CNS immune system. Astroglia were traditionally considered to play primarily a structural and supportive role, in addition to supplying nutrients to the neurons. Recently, the overshadowed role of astroglia in the CNS has been expanded and appreciated (Stern and Filosa, 2013). Astroglia, with thousands of dendrites and synapses, dynamically communicate with surrounding neurons and other glial cells. Any changes in the environment may result in release of cytokines/hormones and enhanced communication between neurons and glia. When severe chronic threats alter the internal milieu of the brain, microglia and astroglia both respond and perpetuate a rise in inflammation in the CNS that has a profound impact on neuronal activity, and consequently BP (Stern and Filosa, 2013; Araque et al., 2014). In addition, recent studies have confirmed the existence of a lymphatic vascular system in the brain, initiating a breakthrough in the brain immunological field (Louveau et al., 2015). Though the complete function of this meningeal lymphatic vessel system is not fully understood, early roles hypothesized include a drainage system for the CSF and clearance of macromolecules from the brain (Aspelund et al., 2015).

#### The IS in BP regulation

Research linking the IS and cardiovascular system has made great progress in recent decades. By utilizing the recombination-activating gene 1 (RAG1)-deficient mice, which lack the mature T and B cells, the important role of immune activation in development of HTN has been illustrated. One important study demonstrates that abnormalities of vascular function were not observed in the RAG1-deficient mice treated with AngII, while AngII-induced BP elevation was also blunted in this model (Guzik et al., 2007). Genetic mutation of RAG1 in Dahl salt-sensitive rat, a hypertension model that can be induced by high salt diet, also attenuated HTN, but adoptive transfer of T cells can restore these cardiovascular abnormalities, indicating the crucial role of T cells in HTN-associated inflammation (Harrison, 2014). Interestingly, AngII-dependent HTN has also been associated with expansion in the splenic B cells and elevation in circulating IgG. This hypertensive response is partially depleted in B cell-activating factor receptor (BAFFR)-deficient mice, and restored by B cell transfer (Chan et al., 2015). These studies clearly demonstrate the essential roles of both T and B cells in different forms of HTN.

As a consequence of an imbalanced local IS, excessive inflammatory mediators from the gut translocate via blood and lymphoid fluid, which may potentially result in inflammation of the CNS (Varatharaj and Galea, 2017). Neuroinflammation, in turn, can result in dysregulated ANS leading to exacerbated neurogenic HTN (Zubcevic et al., 2011). Overactivation of the central IS also leads to production of a variety of inflammatory cytokines and reactive oxygen species (ROS) that subsequently result in damage of the BBB (Yenari et al., 2006), infiltration of inflammatory cells (Gurney et al., 2006) and increased activity in cardioregulatory regions of the brain (Shi et al., 2014). Recently, Santisteban et al. reported that a transplant of bone marrow from the hypertensive SHR to normotensive WKY rats induced activation of peripheral and central inflammation, as well as increased BP in the hypertensive bone marrow chimera (Santisteban et al., 2015). The pro-hypertensive effects of the bone marrow also extended to increase in the sympathetic drive. Thus, overactive peripheral IS can affect the central IS and modulate neuronal activity in cardioregulatory brain regions thus contributing to HTN.

### Role of short chain fatty acids (SCFAs) and lactate in the gut-brain axis

SCFAs, primarily generated by the gut microbiota through anaerobic fermentation, have been shown to have multiple beneficial effects to the host (Hara et al., 1999; Gao et al., 2009; Canani et al., 2011). Acetate, propionate and butyrate are the main metabolic SCFAs generated in the intestine. SCFAs are also present in circulation (Cummings et al., 1987), and are detected in the brain (Kim et al., 2013; Liu et al., 2015). Thus, the presence or absence of SCFAs in circulation may also affect the CNS (Frost et al., 2014; Bourassa et al., 2016). SCFAs bind to the metabolite-sensing receptors, mainly G-protein coupled receptor (Gpr) 41, 43, 109a, and olfactory receptor (Olfr) 78 in mice (homology with Olfr59 in rats) to trigger intracellular signaling. These receptors are widely expressed in diverse organs/tissues, including sympathetic ganglia, endothelial cells, epithelial cells, renal juxtaglomerular apparatus, and smooth muscle cells (Pluznick et al., 2013; Li et al., 2014; Nøhr et al., 2015). Thus, this interaction offers a novel mechanism by which microbial metabolites in the gut can affect BP.

#### SCFAs and BP

SCFAs are vasodilators and thus reduce BP in both rodents (Nutting et al., 1991) and humans (Mortensen et al., 1990) when applied systemically. Intake of dietary fiber, which is the major source of SCFAs *in vivo* after anaerobic fermentation, had positive effects on BP in two separate randomized clinical trials (He et al., 2004; Whelton et al., 2005). Pluznick et al. demonstrated that propionate is able to induce hypotensive responses when administrated in wild type anesthetized mice (Pluznick et al., 2013) in a dose-dependent manner. This BP-lowering effect of SCFAs is differentially modulated by disruption of Olfr78 and Gpr41 gene expression, suggesting there are opposing roles for Olfr78 and Gpr41 in SCFA-mediated BP regulation. Olfr78 was proposed to increase BP, while Gpr41 decreased BP when bound by SCFA (Pluznick, 2014). The mechanisms related to these opposite effects are attributed to the distinct G protein α-subunits and second messengers associated with Olfr78 and Gpr41 receptors (Saito et al., 2009).

#### Role of butyrate in gut-brain axis

Among the major SCFAs, butyrate is the most widely studied. The effects of butyrate on gut-brain axis may be exerted through its impact on the IS, regulation of metabolism, and direct effect on the nervous system.

Its role in modulating IS responses includes, but is not limited to, regulation of recruitment of circulating leukocytes to inflammatory sites, suppression of production of pro-inflammatory cytokines, and modulation of production and release of chemokines and expression of adhesion molecules in neutrophils (Vinolo et al., 2011; Vieira et al., 2012). Moreover, supplementation of butyrate in drinking water of rodents enhances the expression of Foxp3 gene and induces production of regulatory T cells *in vivo*, thus suppressing inflammation (Furusawa et al., 2013). The anti-inflammatory properties of butyrate may also be reflected in its role in epigenetic modification. Butyrate is a potent histone deacetylase (HDAC) inhibitor, and in turn contributes to hyperacetylation of histones and transcription factors. The direct result of this hyperacetylation is ultimate bidirectional changes in transcript expression of downstream genes (Rada-Iglesias et al., 2007; Yang et al., 2013). Since this is a reversible modification in contrast to the genetic defect (Allis and Jenuwein, 2016; Wang et al., 2017), it highlights the potential of butyrate in novel therapeutics. Moreover, HDAC inhibition also exerts anti-inflammatory effects by suppressing the activation of nuclear factor κB (NFκB), a major downstream factor in multiple inflammatory signaling pathways (Adcock, 2007). Another beneficial property of butyrate is its ability to modify the acetylation levels of Foxp3 promoter and thereafter activate the expression of Foxp3 gene in T cells, which is essential for regulatory T cell differentiation (Furusawa et al., 2013). Due to these beneficial effects on the IS, applications for butyrate are actively being pursued and evaluated in immune diseases such as IBD (Tedelind et al., 2007).

Administration of butyrate in diet at 5% wt/wt has also been shown to efficiently increase insulin sensitivity and reduce adiposity (Gao et al., 2009), suggesting that there are metabolic effects of this SCFA. Beneficial metabolic effects of butyrate may extend to direct effects on mitochondrial bioenergetics. In the periphery, butyrate has been shown to increase mitochondrial respiration and energy expenditure (Gao et al., 2009). Moreover, *ex vivo* incubation of butyrate with colonocytes from germ-free (GF) mice rescued the deficits in mitochondrial respiration and inhibited energy deprivation-driven autophagy (Donohoe et al., 2011). In the CNS, the role of astrocytes in glial-neuronal communication is highlighted by their reported ability to donate mitochondrial fragments to neurons, thus favoring the recovery of neurons from ischemia-induced oxidative stress (Hayakawa et al., 2016). Thus, improvement in astrocyte mitochondrial function may be potentially beneficial to neurons in HTN (Hayakawa et al., 2016).

In addition, peripheral butyrate can be detected directly by the butyrate-sensing receptors on afferents (Lal et al., 2001). These afferent nerve responses are abolished in vagotomized rats, indicating the involvement of vagal afferents in the butyrate-responsiveness (Lal et al., 2001).

Thus, the potential impact of butyrate on epigenetic and immunoregulatory mechanisms warrants attention, as these regulatory mechanisms may lead to more specific and efficacious therapeutic strategies for prevention and treatment of different diseases ranging from genetic/metabolic conditions to neurological degenerative disorders (Fernandes et al., 2014; Bourassa et al., 2016).

#### Lactate in gut-brain axis

Previously, we demonstrated a significant increase in lactate-producing bacteria in the hypertensive rodent models (Yang et al., 2015). Moreover, increased concentration of lactate in stool samples from patients with ulcerative colitis or short bowel syndrome has been reported (Vernia et al., 1988; Mayeur et al., 2013). Lactate is primarily fermented to SCFAs by human gut microbiota (Bourriaud et al., 2005). In addition, the environmental pH in the gut also plays an important role in the determination of the capability of microbes to utilize lactate (Belenguer et al., 2007). Accumulation of lactate results in lower pH in the stool, which in turn limits the utilization and conversion of lactate to SCFAs. Therefore, imbalance of lactate and SCFAs could potentially lead to HTN (Demartini et al., 1965; Wikander et al., 1995; Shantha et al., 2013).

Lactate transporters (monocarbohydrate transporters, MCTs) are widely expressed in the intestine and brain, indicating the accessibility of lactate in both organs (Bergersen, 2015). Interestingly, injection of L-lactate into locus coeruleus (LC) induced a significant increase in arterial blood pressure *in vivo* through its excitatory effect on LC (Tang et al., 2014).

### Vagal pathway in gut-brain axis

The vagus nerve is comprised of approximately 90% of afferent fibers that convey sensory information from the periphery to the CNS (Berthoud and Neuhuber, 2000). The nucleus of the solitary tract (NTS) is the major site in the medulla that receives afferent information from visceral organs including the gut. The dorsal nucleus of vagus in the medulla, by contrast, primarily sends output to the gut. In this section, we focus on the vagal signaling in the gut-brain axis.

#### Vagal afferent arm

The cell bodies of visceral vagal afferent neurons are located in the nodose ganglia. The vagal afferent fibers are present within the lamina propria and crypts of GI-tract, from where they relay afferent sensory information to the CNS. In this way, the sensory receptors (chemical and mechanical) present on the vagal afferents can sense local changes in GI homeostasis (Goehler et al., 2005; Cailotto et al., 2012). This information is relayed and informs the CNS on mechanical distension of the intestine, changes in chemicals/pH in the gut, and inflammatory status of the tissue. In view of the latter, it has been shown that peripheral administration of endotoxin lipopolysaccharide (LPS) or IL-1β can induce activation of vagal afferents in the gut (Goehler et al., 2000; Pavlov and Tracey, 2012). This mechanism is dominant when intestinal inflammatory cytokines are undetectable in circulation by CVOs during a low-grade inflammation. Moreover, presence of Toll-like receptor 4 (TLR4) within the nodose ganglia also supports a potential role of vagal afferents in sensing the systemic immunoactive molecules in addition to localized intestinal inflammation (Hosoi et al., 2005).

As part of the afferent limb of the vagal pathway, the NTS plays an essential role in receiving the vagal afferent information (Pavlov and Tracey, 2012). Glutamate is the major neurotransmitter conveying information from the ascending vagal afferents to NTS. The secondary neurons in NTS that detect the afferent glutamatergic input form a tight network of glutamatergic and GABAergic (gamma-amino butyric acid-releasing) neurons, processes the incoming afferent signals and subsequently projects it to other brain regions as well as to downstream cholinergic efferent neurons modulating peripheral responses. This signal relay eventually results in either excitatory or inhibitory effects on the gut (Travagli et al., 2003) as well as the cardiovascular system and the IS (Mancia and Grassi, 2014).

#### Vagal efferent arm

Vagal efferents innervate a number of organs, including the gut. The direct communication between the enteric nervous system (ENS) and CNS is mediated via the vagus nerve. In this way, the CNS monitors the homeostatic state of the GI tract and regulates contractile properties and acid secretion through the vago-vagal reflex. In contrast, the ENS preserves complete reflex circuits (sensors-interneuron-motor neurons). Therefore, intestinal contractile/distension, local blood flow and nutrient absorption is regulated locally within the intestine. Removal of the vago-vagal reflex, thus, has minor impacts on the overall intestinal function (Furness et al., 2014).

#### Vagal pathway in BP regulation

Selective vagal nerve stimulation has been shown to lower BP by reducing heart rate (Gierthmuehlen and Plachta, 2016). The beneficial effects of vagal nerve stimulation extend to the reduction of intestinal, as well as systemic inflammation (Matteoli and Boeckxstaens, 2013; Koopman et al., 2016). Thus, considering the diminished vagal properties in HTN, there may be multiple benefits of activation of vagal efferents in HTN, including direct dampening of the IS responses. It has not been discussed whether the impacts of gut dysbiosis on BP may be through vagal pathway. However, multiple gut peptides can be sensed by vagal sensory neurons (Grabauskas and Owyang, 2017). In addition, an association between obesity and altered vagal pathway has been established, characterized by the reduced mechanical sensitivity in the jejunum (Daly et al., 2011) and reduced c-fos expression in the AP and NTS upon CCK treatment (Covasa et al., 2000).

### Endocrine systems and neurotransmitters in gut-brain axis

#### Angiotensin II (AngII)

AngII is a vasoactive peptide of the RAS that can raise the BP via direct vasoconstriction, activation of SNS, activation of IS, and induction of biosynthesis of aldosterone. Two distinct but interconnected parts of RAS (peripheral and central) can contribute to elevation of BP via both independent and interdependent mechanisms, whereby the CVOs bridge the connection between peripheral and central AngII effects. We have recently demonstrated the presence of gut dysbiosis and gut inflammation in AngII-induced HTN, also characterized by dysfunctional ANS and central inflammation (Yang et al., 2015; Santisteban et al., 2017). However, it is not clear whether the GI/microbiota effects are a cause or consequence in the AngII-induced HTN, and whether there is a prominent role of AngII in modulating the microbiome. Recently, it has been demonstrated that AngII HTN and vascular dysfunction are blunted in GF mice (Karbach et al., 2016), suggesting that gut microbiota may contribute to the AngII-induced BP increase.

#### Serotonin (5-hydroxytryptamine, 5-HT)

Serotonin is a monoamine neurotransmitter derived from tryptophan. It is primarily found in the GI tract, in blood platelets, and in the CNS (Yano et al., 2015). More than 90% of the 5-HT in our body is synthesized in the gut, and 5-HT either diffuses into circulation where it is sequestered by platelets, or it binds to its receptors that are widely distributed on enteric neurons, enterocytes, and immune cells (Watts et al., 2012). Although it is generally accepted that 5-HT cannot translocate from peripheral circulation into the brain across the BBB, it has been suggested that alterations in gut microbiota have effects on 5-HT levels in the hippocampus (Diaz Heijtz et al., 2011), and that endothelial cells in the brain actively express 5-HT transporters (Nakatani et al., 2008). In addition, the presence of 5-HT receptors in the CVOs may also mediate the connection between gut and brain (Takeuchi and Sano, 1983; Scrogin et al., 1998).

Notably, serotonin has been associated with pulmonary HTN due to the discovery that anorexigens, indirect serotonergic agonists, can cause pulmonary arterial HTN (MacLean and Dempsie, 2009). The potential mechanisms contributing to pulmonary arterial HTN include increased expression of 5-HT receptors, reduction in serotonin transporter (SERT), and generation of reactive oxygen species (ROS) in the lung (MacLean and Dempsie, 2009). The role of serotonin in BP control has been reviewed elsewhere (Watts et al., 2012). Interestingly, the dysregulated production of serotonin in anxiety and depression may also contribute to increased BP (Frick et al., 2015). Psychosocial stressors associated with anxiety disorders elicit activation of ANS and HPA axis, which consequently predisposes individuals to the likelihood of developing HTN (Player and Peterson, 2011).

#### GABA, glutamate and dopamine

GABA is a major inhibitory neurotransmitter in the mammalian CNS. GABAergic neurons are present and involved in regulation of excitation of several cardioregulatory brain regions, and modulation of vagal signaling within the NTS. Elevated GABA signaling in the NTS has been associated with HTN (Li et al., 2013) and diabetes (Boychuk and Smith, 2016). GABA is also an essential cardioregulatory neurotransmitter in the paraventricular nucleus (PVN) of hypothalamus, where it reportedly contributes to determining the level of sympathetic outflow. For example, microinjection of a GABA antagonist into the PVN produced significant dose-dependent increase in renal sympathetic nerve activity (Li et al., 2006), suggesting inhibitory modulation on the pre-sympathetic PVN neurons.

Glutamate is a major excitatory neurotransmitter in the CNS. Activation of vagal afferents results in the release of glutamate in the NTS and generates changes in membrane potentials of the second-order NTS neurons by binding to alpha-amino-3-hydroxy-5-methyl-4-isoxazole proprionic acid (AMPA) receptors or N-methyl-D-aspartate (NMDA) receptors, which can contribute to the maintenance of resting membrane potential or regulate convergence of stimulatory inputs, respectively (Bonham and Chen, 2002). Injection of glutamate into NTS produces dose-dependent hypotension (Talman et al., 1984), in line with the role of glutamate in baroreceptor reflex responses. On the other hand, microinjection of glutamate into the PVN produced a dose-dependent increase in renal sympathetic nerve activity and BP, effects that can be blocked by the NMDA receptor antagonist (Li et al., 2006).

Both GABA and glutamate have been shown to be abundant in the intestine (Reeds et al., 2000; Hyland and Cryan, 2010), and the GI tract harbors abundant gram-positive facultative anaerobic bacteria *Lactobacillus* and *Bifidobacterium*, both of which are able to metabolize glutamate to produce GABA (Boonstra et al., 2015). The results of several studies support two basic pathways through which GI-derived GABA can be sensed and utilized by the CNS: (i) GI-derived GABA may be able to diffuse into circulation and cross the BBB (Takanaga et al., 2001; Steenbergen et al., 2015a); and (ii) GI-derived GABA can be sensed by GABA receptors within the ENS, which directly communicates with vagal afferents (Auteri et al., 2015; Steenbergen et al., 2015b). However, direct evidence for these is still lacking to reach a firm conclusion.

Dopamine (D), a common neurotransmitter, is produced in both neuronal and nonneuronal cells. Previous study suggested that almost half of D in the body was produced in the GI tract (Eisenhofer et al., 1997). The locally produced D (i.e., renal proximal tubule, jejunum, *Bacillus cereaus, B. mycoides, B. subtillis* Zeng and Jose, 2011; Clark and Mach, 2016) is independent of innervation, and has shown significant effects on BP regulation via renal D1-like receptors that modulate NaCl excretion (Zeng and Jose, 2011). Long term treatment of D1-like receptor antagonist increased BP, and impairment of renal D1-like receptor has been associated with HTN (Haney et al., 2001). Another important D receptor, D3, plays a significant role in natriuresis and diuresis. D3^−/−^ and ^−/+^ mice exhibit higher systolic and diastolic BP compared with wild type controls. SHR, characterized by reduced expression of D3 receptors, shows resistance to the BP-lowering effects of selective D3 agonists.

## Interplay of IS, SNS and gut dysbiosis in HTN

Role of SNS in control of BP and HTN has been studied extensively (Mancia and Grassi, 2014; Zubcevic et al., 2014). Interventions modulating renal sympathetic activity are shown to be efficient in BP control (Xiao et al., 2015), though this innovative technology should be used with caution before the evaluation of long-term safety and efficacy data are completely obtained (Fengler et al., 2016). Moreover, denervation of splenic sympathetic activity can prevent T cell activation and egression from the spleen (Carnevale et al., 2016). In addition, our group recently demonstrated that the loss of sympathetic signaling in the bone marrow decreased BP and suppressed systemic and gut immune responses (Eberle et al., 2014; Ahmari et al., 2016). Thus, a reciprocal IS-SNS communication exists in which a deregulation of one can lead to dysfunction of other in HTN. We have recently shown that the suppressed immune responses in the gut, as a function of reduced SNS effects on the IS, also produced beneficial alterations in the gut microbiota (Yang et al., 2017). Therefore, changes in the SNS activity have impacts on BP, which is associated with alterations in the IS and gut microbiota.

Since the initial observation suggesting a link between gut dysbiosis and HTN in 2015(Mell et al., 2015; Yang et al., 2015), a few new studies have further investigated this interaction. Recently published data demonstrated that fecal transplantation from hypertensive rats and human patients induced BP increase in normotensive rats and mice, respectively (Adnan et al., 2017; Li et al., 2017a), indicating a causative and/or contributory role for gut dysbiosis in HTN. However, these studies remain descriptive and the precise mechanisms behind the association between gut dysbiosis and HTN remain elusive. Considering the complexity of interactions and vastness of potential mediators as reviewed in the present manuscript, future studies should attempt to elucidate mechanistic interactions between microbial, neuronal and IS effectors in health and HTN.

## Concluding remarks

The prevalence of HTN and its debilitating role as a leading risk factor for premature death, stroke and heart diseases have expanded in the past decade. Projections show that around 41.4% of US adults will have HTN by 2030 (Heidenreich et al., 2011). Therefore, it is imperative to develop an effective treatment and/or prevention strategy to reduce the burden of HTN, especially resistant HTN, of which the available treatments have been largely ineffective. The recent associative link between the gut dysbiosis and HTN has opened the floodgates in research on the role of the gut microbiome in CVD. We propose an integrated network that regulates BP that involves feedback between IS, nervous system and gut microbiota. Naturally, manipulation of gut microbiota may have distinct advantages, offering the possibility of relatively non-invasive and inexpensive therapeutics. However, considering the complexity of interplay between the three systems, manipulation of one may not be sufficient in fully relieving the effects of the disease. All said, the advancement in our knowledge on the role of gut microbiota in CVD would greatly favor ~970 million people worldwide.

## Author contributions

All authors listed, have made substantial, direct and intellectual contribution to the work, and approved it for publication.

### Conflict of interest statement

The authors declare that the research was conducted in the absence of any commercial or financial relationships that could be construed as a potential conflict of interest.
